# Suppressing STAT5 signaling affects osteosarcoma growth and stemness

**DOI:** 10.1038/s41419-020-2335-1

**Published:** 2020-02-24

**Authors:** Dharmalingam Subramaniam, Pablo Angulo, Sivapriya Ponnurangam, Prasad Dandawate, Prabhu Ramamoorthy, Pugazhendhi Srinivasan, Tomoo Iwakuma, Scott J. Weir, Katherine Chastain, Shrikant Anant

**Affiliations:** 1 Department of Cancer Biology, The University of Kansas Medical Center, Kansas City, KS 66160 USA; 2 Division of Hematology and Oncology, Children’s Mercy Hospital, Kansas City, MO 64108 USA; 3 Present Address: Banner Health, 1432S. Dobson Rd. Ste. 107, Mesa, AZ 85202 USA; 4 Present Address: Janssen Inc, 1000 U.S. Route 202 South, Raritan, NJ 08869 USA

**Keywords:** Targeted therapies, Sarcoma

## Abstract

Osteosarcoma (OS) is the most common primary bone tumor that primarily affects children and adolescents. Studies suggested that dysregulation JAK/STAT signaling promotes the development of OS. Cells treated with pimozide, a STAT5 inhibitor suppressed proliferation and colony formation and induced sub G0/G1 cell cycle arrest and apoptosis. There was a reduction in cyclin D1 and CDK2 expression and Rb phosphorylation, and activation of Caspase-3 and PARP cleavage. In addition, pimozide suppressed the formation of 3-dimensional osteospheres and growth of the cells in the Tumor in a Dish lung organoid system. Furthermore, there was a reduction in expression of cancer stem cell marker proteins DCLK1, CD44, CD133, Oct-4, and ABCG2. More importantly, it was the short form of DCLK1 that was upregulated in osteospheres, which was suppressed in response to pimozide. We further confirmed by flow cytometry a reduction in DCLK1+ cells. Moreover, pimozide inhibits the phosphorylation of STAT5, STAT3, and ERK in OS cells. Molecular docking studies suggest that pimozide interacts with STAT5A and STAT5B with binding energies of −8.4 and −6.4 Kcal/mol, respectively. Binding was confirmed by cellular thermal shift assay. To further understand the role of STAT5, we knocked down the two isoforms using specific siRNAs. While knockdown of the proteins did not affect the cells, knockdown of STAT5B reduced pimozide-induced necrosis and further enhanced late apoptosis. To determine the effect of pimozide on tumor growth in vivo, we administered pimozide intraperitoneally at a dose of 10 mg/kg BW every day for 21 days in mice carrying KHOS/NP tumor xenografts. Pimozide treatment significantly suppressed xenograft growth. Western blot and immunohistochemistry analyses also demonstrated significant inhibition of stem cell marker proteins. Together, these data suggest that pimozide treatment suppresses OS growth by targeting both proliferating cells and stem cells at least in part by inhibiting the STAT5 signaling pathway.

## Introduction

Osteosarcoma (OS) is the most common pediatric bone malignancy in the world and the eighth most common childhood cancer. Incidence of OS is 4.4 per million per year from birth to 24 years of age^1^. While only 20% of patients present with metastasis that is clinically detectable, the majority of the remaining 80% are presumed to have undetectable micrometastases at diagnosis, primarily in the lung^2^. The five-year overall survival for metastatic OS ranges from 20–40%. Current therapy includes surgery and chemotherapy. Traditional chemotherapeutic agents have included high dose methotrexate, cisplatin, doxorubicin, and ifosfamide^3^. While chemotherapy has increased overall survival in localized OS, survival rates have remains stagnant for the last 30–40 years^4^. Moreover, only 30% of patients with metastatic OS achieve long-term survival. For those who have recurrent disease, prognosis is poor with post-relapse survival of less than 20%^5^. Due to limited success of surgical resection and systemic chemotherapy for metastatic OS, there is a need to evaluate new treatment regimens that could potentially offer increased cure and survival in these afflicted patients.

**Fig. 1:**
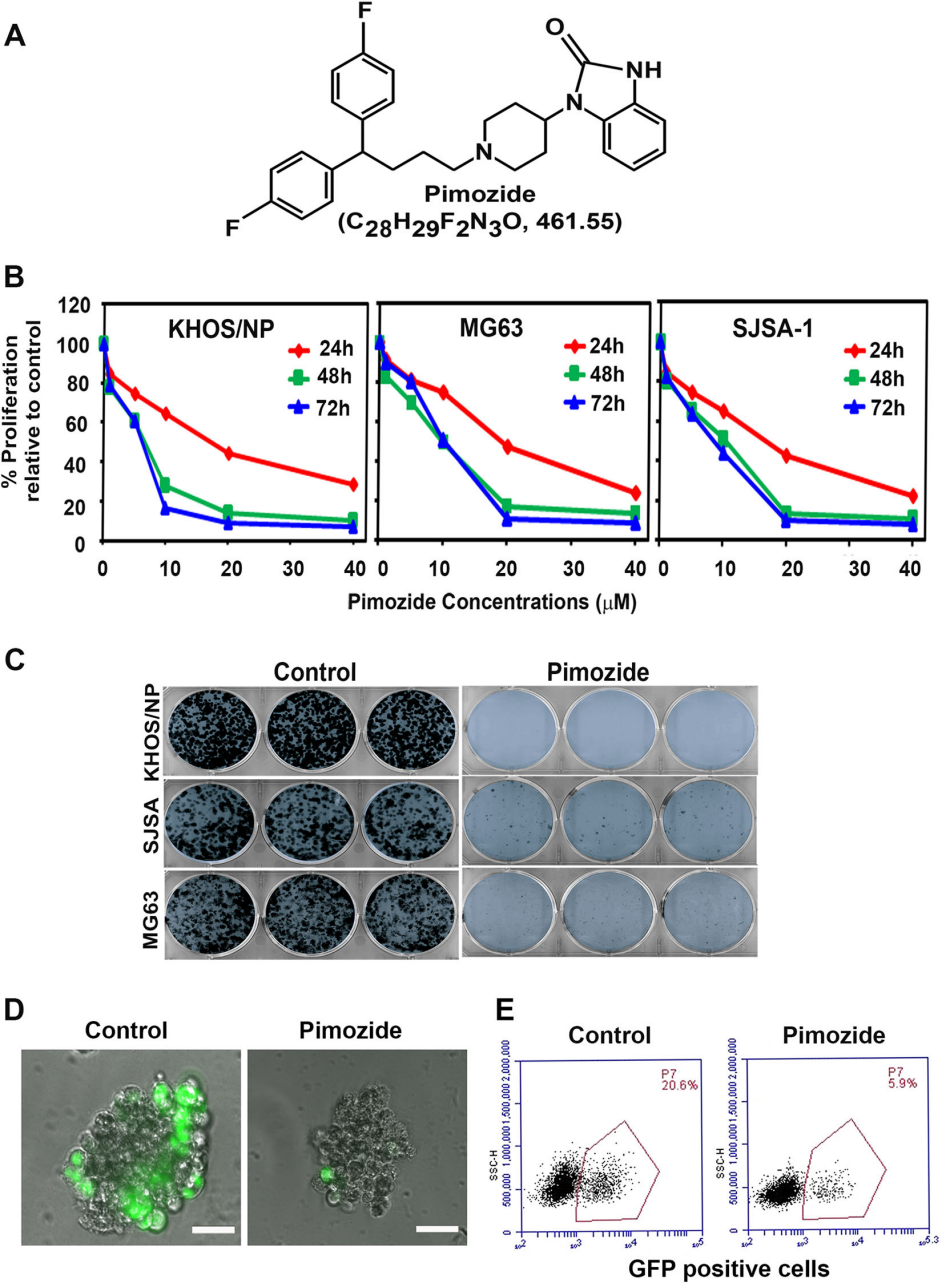
Pimozide inhibits osteosarcoma growth. **a** Pimozide structure. **b** KHOS/NP, MG63, and SJSA-1 cells were incubated with increasing doses of pimozide (0–40 μM), and cell proliferation was determined. Pimozide treatment resulted in a significant dose-and time-dependent decrease in cell proliferation in all cell lines when compared with controls. **c** KHOS/NP, MG63, and SJSA-1 cells were incubated with 10 µM pimozide for 48 h and the cells were allowed to grow and form colonies. Pimozide treatment inhibits colony formation. Results are representative of three independent experiments. **d** and **e** Pimozide at 20 μM concentration specifically inhibits the growth of KHOS/NP GFP positive cells, but not the normal cells in the TiD system. Scale bars represent 100 μm.

Studies speculate that the antipsychotic medications may also provide antineoplastic effects against cancers. Pimozide (Fig. 1a) is an FDA-approved cell-permeable and orally available drug belonging to the diphenylbutylpiperidine class that has been regularly used for treating schizophrenia and Tourette syndrome^6^. In the anterior pituitary, the compound functions by binding to the dopamine receptor D2 resulting in a decrease in the dopamine neurotransmission and serum elevation of the hormone prolactin^7^. Pimozide has also demonstrated the ability to inhibit cancer growth in neuroblastoma, lymphoblastoma, breast cancer, and non-small cell lung cancers^8^.

Janus Kinases (JAK) are a family of cytoplasmic tyrosine kinases essential in the signaling cascade for cytokines and growth factors^9^. Upon being phosphorylated, JAK can activate a family of transcription factors known as signal transducer and activator of transcription (STAT). Upon being phosphorylation, STAT will translocate from the cytoplasm to the nucleus where it can mediate transcription of target genes involved in proliferation, differentiation, and survival^10^. Pimozide has been shown to inhibit the constitutive STAT5 Tyr694 phosphorylation, although the mechanism of this inhibition is currently unknown^10,11^.

Recent studies supporting the presence of cancer stem cells (CSCs) have led to efforts to identify and develop therapeutic strategies targeting these cells^12^. In addition, it has been shown that CSCs are highly resistant to standard therapy^13^. Stemness-related markers in OS are ABCG2, Nestin, CD44, STRO-1, and Endoglin/CD105^14–17^. Previously, we identified a novel protein doublecortin calmodulin-like kinase 1 (DCLK1) that is present in reserve quiescent stem cells in both the normal intestinal epithelium and in colon cancers^18,19^. Moreover, the majority of human colon cancers was shown to express the short form of DCLK1^20^. Studies confirmed that DCLK1 distinguishes between tumor and normal stem cells in the intestine and could be therapeutic target for colon cancer^21,22^. In OS, mesenchymal stem cells have been shown to induce IL-6 expression, which then activates the JAK/STAT signaling pathway by binding to their cognate receptor in OS cells. Moreover, blocking STAT3 activation using an IL-6 neutralizing antibody resulted in decreased cell proliferation, migration, and cell death of the cancer cells^23^. Studies suggested that blocking the JAK/STAT pathway is therefore a potential option for OS treatment^24,25^. One study has suggested that targeting growth hormone stimulated STAT5 activation results in decreased STAT5 transcriptional activity^26^. In this report, we have determined the effect of pimozide on STAT5, and on OS cell growth and stemness.

## Results

### Pimozide inhibits OS cell growth

We first determined the effect of pimozide (Fig. 1a) on proliferation of various cultured OS cell lines. Pimozide inhibits proliferation of KHOS/NP, MG63, and SJSA-1 in a dose and time dependent manner (Fig. 1b). This anti-proliferative effect on the OS cells was initially seen within a 24 h period, and this effect continued to significantly increase over the next 72 h. The inhibitory constant value at 48 h in KHOS/NP, MG63, and SJSA-1 cell lines was determined to be 8.5, 10, and 9 µM, respectively (Fig. 1b). To determine the long-term effect of pimozide treatment, we treated the OS cells with 10 µM pimozide for 48 h. Thereafter, the pimozide was removed and the cells were allowed to grow and form colonies. Treatment with pimozide resulted in significantly reduced number of colonies in all three OS cell lines (Fig. 1c). This suppression suggests the irreversible effect of pimozide on the OS cells. To further demonstrate that the compound affects OS cells and not normal cells, we performed studies in the multicell type lung organoid model (called TiD)^27^. The TiD organoid contains immortalized lung epithelial and fibroblasts, along with endothelial cells thereby creating a in vivo-like tumor microenvironment providing the necessary cell-cell contact, architecture, and influence of different cell types for the OS cells^27^. We generated TiDs with OS cells which were labeled with GFP. When the TiD was treated with 20 µM pimozide, there was a significant reduction in growth of the OS cells without affecting the normal cells (Fig. 1d, e). These data suggest the compound affects growth of OS cells, without affecting normal cells.

### Pimozide induces G0/G1 cell cycle arrest and apoptosis in OS cells

**Fig. 2:**
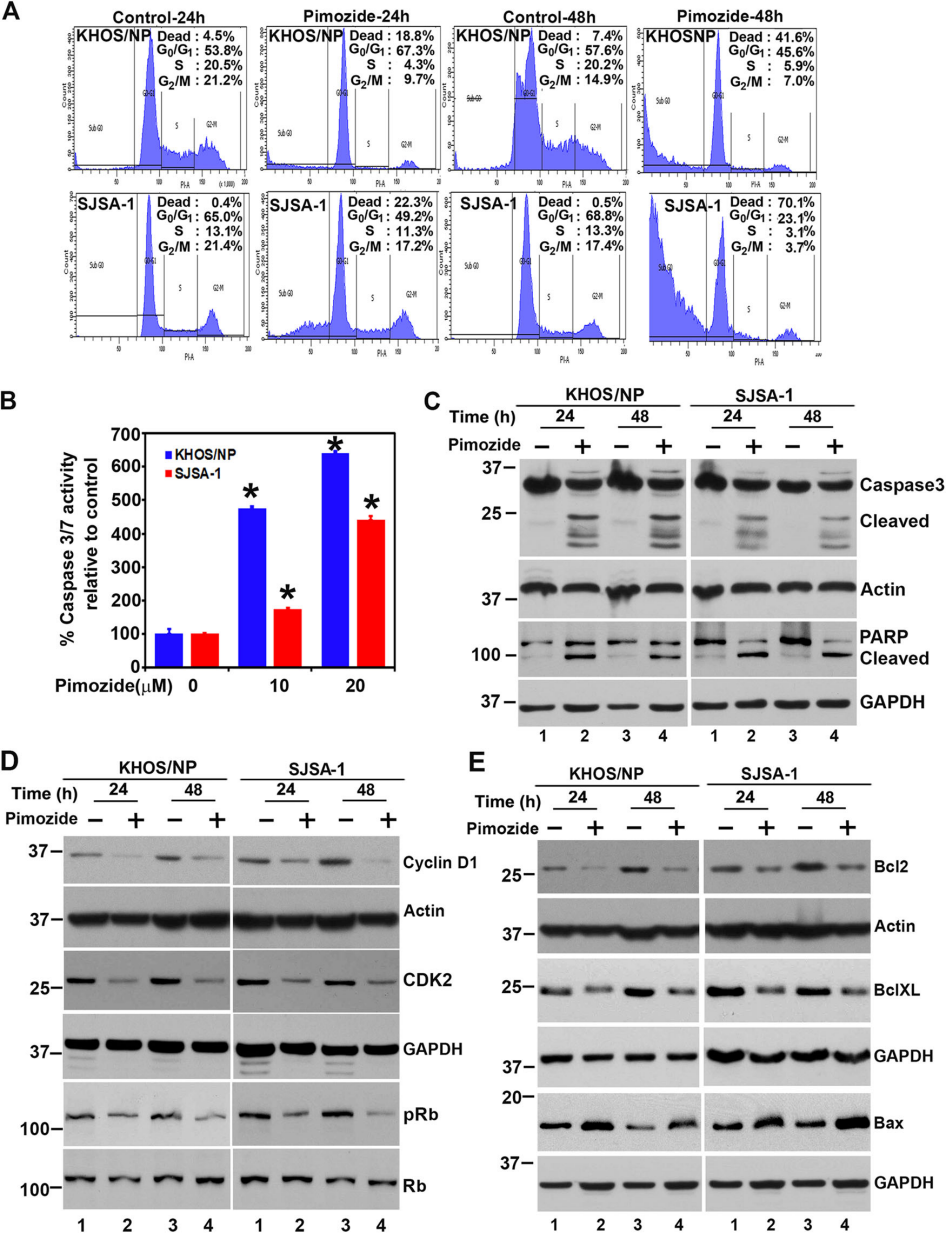
Pimozide induces cell cycle arrest and apoptosis. **a** Cell cycle analysis of pimozide treated cells. KHOS/NP and SJSA-1 were treated with 20 µM of pimozide for 24 and 48 h and then examined by flow cytometry following propidium iodide staining for DNA content. Pimozide treatment increased SubG0/G1 arrest at 24 and 48 h in both cells. **b** Cells were incubated with pimozide 10 and 20 µM concentration for 24 h and assessed for apoptosis by Caspase3/7 assay. Pimozide treatment results in significant increase in caspase-3/7 activity in both KHOS/NP and SJSA-1 cells (*p < 0.001). **c** Cells were treated with 20 μM of pimozide for 24 and 48 h, the lysate was analyzed by western blotting for Caspase 3 and PARP protein expression. Both the cell lines showed caspase 3 cleavage and PARP cleavage following pimozide treatment. **d** Lysates from cells incubated with 20 µM of pimozide were analyzed by western blotting for cyclin D1, along with CDK2 and phosphorylation of RB levels. Pimozide inhibits cyclin D1, CDK2 expressions and phosphorylation of RB levels in both KHOS/NP and SJSA-1 cells. **e** Cells wer**e** treated with 20 μM of pimozide for 24 and 48 h, the lysate was analyzed by western blotting for anti-apoptotic and pro-apoptotic proteins. Pimozide treatment resulted in decreased levels of Bcl2 and Bcl-XL expression and increased Bax expression in both KHOS/NP and SJSA-1 cells.

Given that the Pimozide inhibits proliferation and colony formation; we next determined whether the compound affects cell cycle progression. For this, we chose two cell lines KHOS/NP and SJSA-1. Treatment with pimozide significantly increased G0/G1 and sub-G0 arrest in both KHOS/NP and SJSA-1 cells (Fig. 2a). This was observed when the cells were treated with pimozide for either 24 h or 48 h. Next, we determined whether pimozide induces apoptosis in OS cell lines. For this, we treated the cells with 10 or 20 µM pimozide for 24 h and 48 h, following which we first determined caspase 3/7 activity using a fluorescence-based assay. Treatment with pimozide significantly increased caspase 3/7 activity in both KHOS/NP and SJSA-1 cells (Fig. 2b, Supplementary Fig. 1a). These results were further confirmed by western blotting for caspase 3 activation and PARP cleavage. Again, pimozide treatment demonstrated increased levels of cleaved caspase 3 and PARP proteins in both KHOS/NP and SJSA-1 cells (Fig. 2c).

Cyclin D1 is a cell cycle regulatory protein that regulates the G1 to S-phase transition of the cell cycle. Cyclin D1 overexpression has been linked to the development and progression of cancer^28^. Pimozide treatment inhibited cyclin D1 expression (Fig. 2d). In addition, we performed western blots to determine levels of the G0/G1 cell cycle marker protein CDK2 and phosphorylation of retinoblastoma protein (Rb). Pimozide treatment significantly reduced CDK2 expression, as well as phosphorylation of Rb in both KHOS/NP and SJSA-1 cells (Fig. 2d). In addition, we also performed western blot for CDK4 and CDK6^29^. Pimozide treatment significantly reduced CDK4 and CDK6 expression in both KHOS/NP and SJSA-1 cells (Supplementary Fig. 1b). We next determined whether the pimozide induces apoptosis by performing western blot analyses for the anti-apoptotic proteins Bcl2 and Bcl-XL, and pro-apoptotic protein Bax. Pimozide reduced Bcl2 and Bcl-XL levels, whereas there was a significant increase in Bax protein levels in both KHOS/NP and SJSA-1 cells (Fig. 2e). These data suggest that pimozide induces apoptosis.

### Pimozide inhibits stemness

**Fig. 3:**
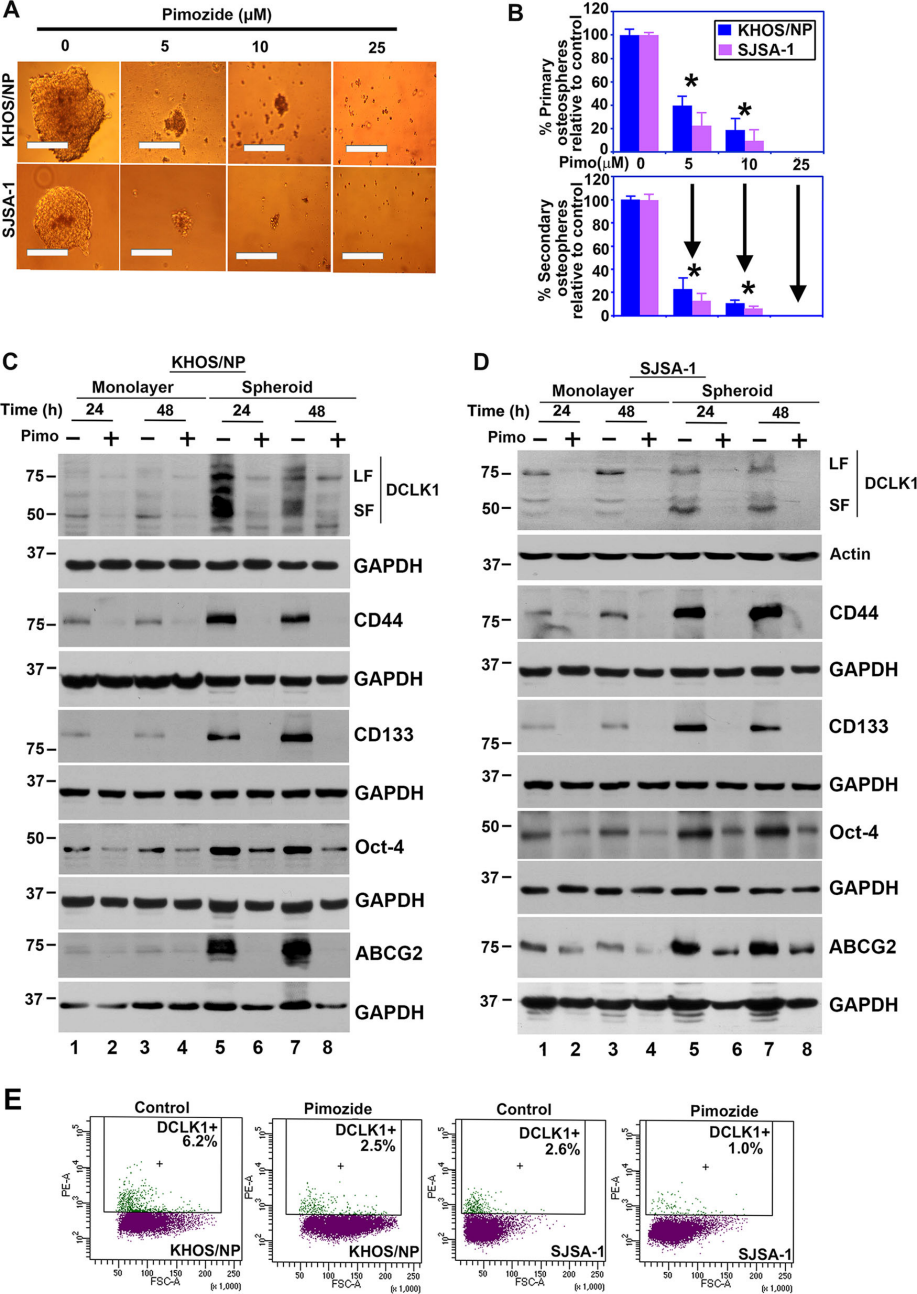
Pimozide affects cancer stem cell marker expression. **a** KHOS/NP and SJSA-1 cells were grown in specific spheroid media in low adherent plates and treated with 0–25 μM of pimozide. After 5 days, the spheroids were photographed and counted. Scale bars represent 100 μm. **b** Primary spheroids were collected and separated into single cells and re-plated. Pimozide treatment significantly inhibited osteosphere formation (*p < 0.05). **c** and **d** Western blot analyses of lysates from 3D cultures showed significant increase of DCLK1 short form in both KHOS/NP and SJSA-1 cells when compared to 2D cultures. Pimozide inhibits the expression of the DCLK1 isoforms. Western blot analyses of lysates from 3D cultures showed significant increase of CD44, CD133, Oct-4, and ABCG2 levels in both KHOS/NP and SJSA-1 cells when compared to 2D cultures which was suppressed by pimozide. **e** Sorting of anti-DCLK1 antibody-tagged phycoerythrin in KHOS/NP and SJAS-1 cells by flow cytometry. After 24 h, pimozide treatment resulted in a significant reduction in the number of DCLK1 positive cells.

Defining the mechanisms that regulate stem cell fate is critical to increase our understanding of the neoplastic process. CSCs are capable of self-renewal and generating tumors resembling the primary tumor^30,31^. We first determined the effects of pimozide on sphere-formation assay, a well-accepted biological property representing stemness. Treatment of OS cells with pimozide significantly inhibited osteosphere formation in KHOS/NP and SJSA-1 cells (Fig. 3a, b top panel). To confirm the reduction was due to loss of stem cells, primary spheroids were dissociated, and the equal number of cells were reseeded without any additional pimozide. There was a further reduction in secondary spheroid formation in the pimozide treatment group (Fig. 3b bottom panel). To confirm the effect on CSCs, we next determined the effect of pimozide on the expression of stem cell marker proteins. Previously, we and others had identified the protein Doublecortin-like kinase 1 (DCLK1) to be a reserve quiescent stem cells in both the normal intestinal epithelium and in colon cancers^18,19^. Further studies confirmed that DCLK1 distinguishes between tumor and normal stem cells in the intestine and could be therapeutic target for colon cancer^21,22^. DCLK1 has been shown be expressed in two forms, a full-length large form that encodes two microtubule-associating DCX domains at the N-terminus and a calmodulin-like kinase domain at the C-terminus. In addition, a shorter variant of the protein is expressed that lacks the N-terminal DCX domain^32–35^. To determine whether DCLK1 is expressed in OS cells, we performed western blot analyses of extracts generated from cells grown in 2-dimensional (2D) monolayer and 3-dimensional (3D) spheroid cultures. There was expression of the long and short form of DCLK1 in both KHOS/NP and SJSA-1 cells (Fig. 3c, d). Furthermore, the two isoforms of the protein were upregulated in 3D osteospheres of both KHOS/NP and SJSA-1 cells (Fig. 3c, d). Moreover, following pimozide treatment, there was greater decrease in the shorter isoform of the protein. Further confirmation of reduction was obtained by flow cytometry demonstrating reduced numbers of DCLK1 + cells (Fig. 3e). Pimozide treatment also reduced the expression of CD44, CD133, Oct-4, and ABCG2 levels in both monolayer and 3D osteospheres cultures in both KHOS/NP and SJSA-1 cells (Fig. 3c, d). Taken together, these data suggest that pimozide treatment affects osteosarcoma stem cells.

### Pimozide inhibits STAT5 signaling

**Fig. 4:**
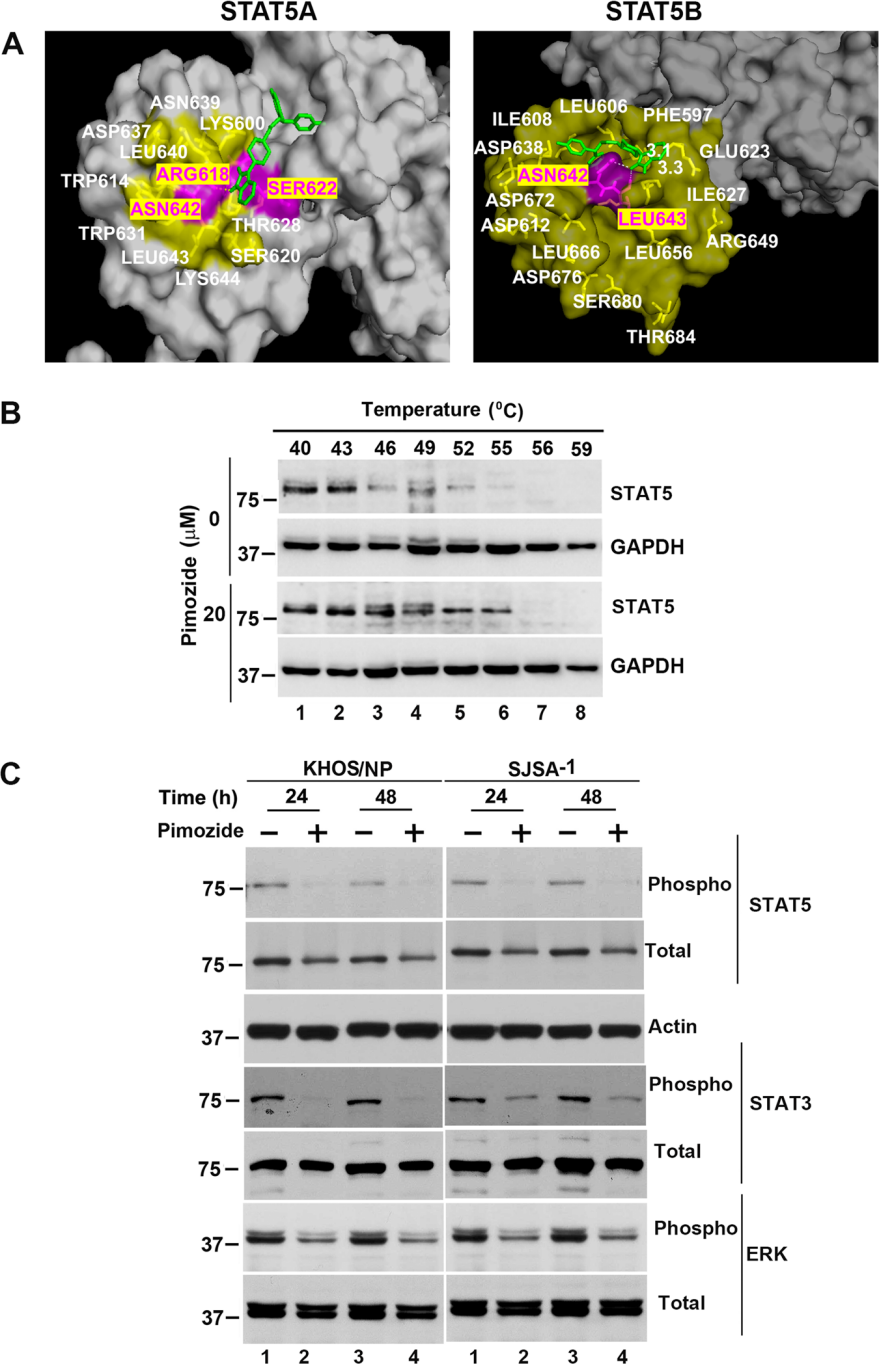
Pimozide inhibits STAT5 phosphorylation. **a** Pimozide interacts with the STAT5. We performed molecular docking and determined that pimozide can interact with the SH2 domain of STAT5A and STAT5B with binding energy of −8.4 and −6.4 kcal/mol, respectively. **b** Cellular thermal shift assay (CETSA). KHOS/NP cells were treated with pimozide and subjected to differential temperature treatment for 3 mins. Resulting lysates were subjected to western blot analyses for STAT5. Pimozide protected STAT5 against thermal denaturation suggesting that pimozide-STAT5 interaction. **c** Lysates from pimozide treated cells caused a significant decrease in the phosphorylation of STAT5, STAT3, and ERK.

**Fig. 5:**
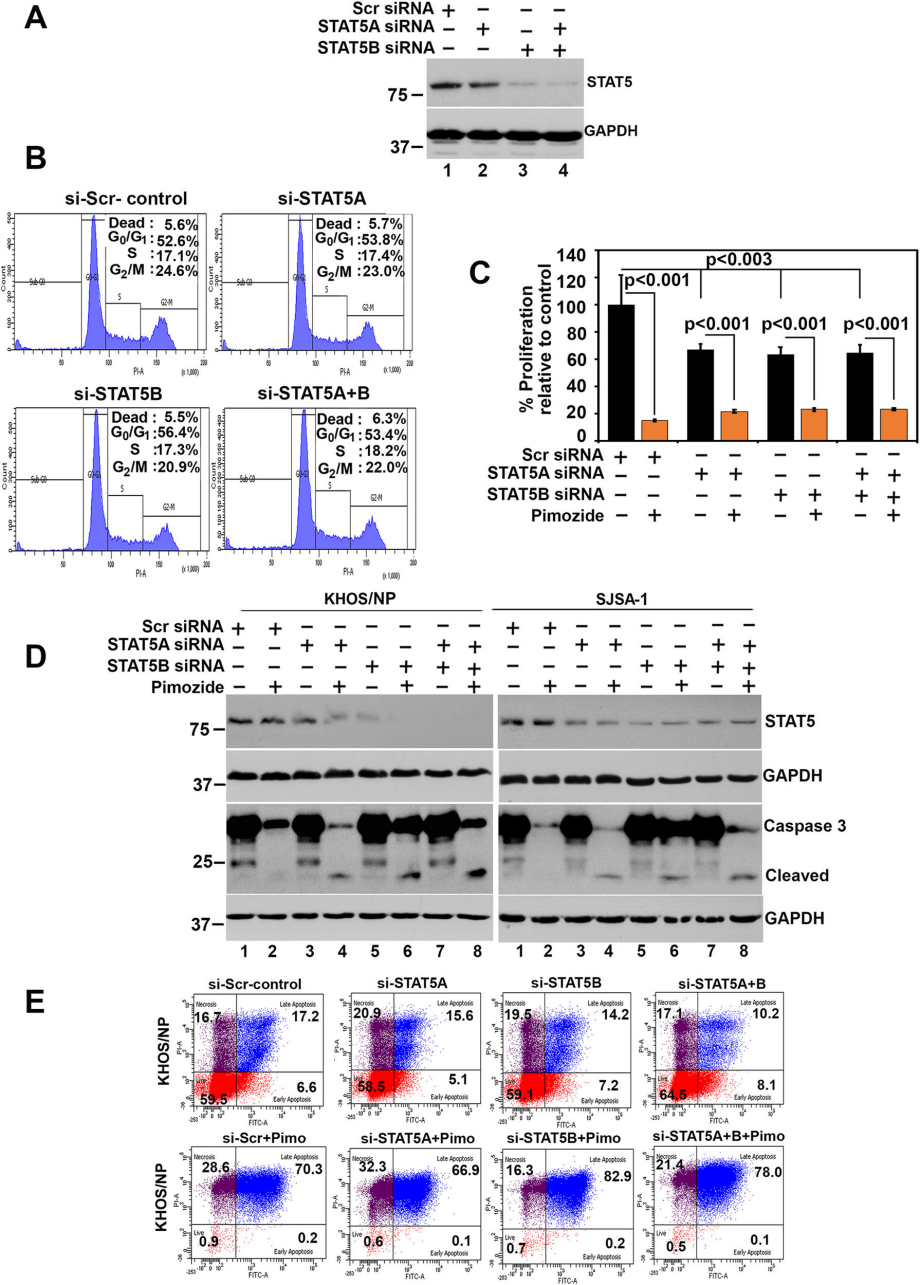
STAT5 protects cells from pimozide-mediated cell death. **a** Cells were transfected with siRNA for STAT5A, STAT5B or both for 72 h. Lysates were analyzed STAT5 western blot. Knockdown of STAT5A or STAT5B reduced STAT5 protein levels, with greater reduction observed in cells transfected with STAT5B. **b** Cells were transfected with siRNA for STAT5A, STAT5B or both for 48 h, and subsequently subjected to flow cytometry following staining with propidium iodide. Knockdown of STAT5 protein did not affect cell cycle progression of OS cells. **c** Cells transfected with siRNA to STAT5A, STAT5B or both were treated with 10 µM pimozide. Cell proliferation measured by hexosaminidase assay demonstrated that knockdown of STAT5 reduced cell proliferation. **d** Cells were transfected with siRNA for STAT5A, STAT5B or both for 48 h and then treated with 10 µM pimozide for 24 h. Lysates were analyzed for STAT5 and Caspase 3 levels. Knockdown of STAT5A or STAT5B further enhanced pimozide mediated caspase 3 cleavage. **e** Cells were transfected with siRNA to STAT5A, STAT5B or both and then treated with pimozide were analyzed by flow cytometry following AnnexinV/PI staining. Pimozide treatment significantly induced cells to undergo necrosis and late apoptosis. However, knockdown of STAT5B reduced pimozide mediated necrosis resulting in further increase in apoptosis.

Previous studies have suggested that pimozide inhibits STAT3 and STAT5 signaling^10,36^. However, the precise mechanism of the inhibition is not known. These STAT genes encode proteins required for self-renewal, cell survival and proliferation^37^. Pimozide inhibits STAT3 phosphorylation resulting in suppression expression of target genes BclXL, Mcl1 and Myc in hepatocellular carcinoma^38^. Previous studies have also shown that although pimozide decreases STAT5 tyrosine phosphorylation, although it does not suppress tyrosine kinases^10^. Using the crystal structure for STAT5A (PDB:1Y1U) and STAT5B (PDB: 6MBW), we performed homology modeling to determine how pimozide interacts with the proteins^39,40^. We chose the region for our studies because the activating phosphorylation site in STAT5A and STAT5B are Tyr694 and Tyr699, respectively^41^. Pimozide demonstrated strong binding to both STAT5A and STAT5B (Fig. 4a). The binding energies for STAT5A and STAT5B were predicted to be −8.4 kcal/mol and −6.4 kcal/mol, respectively (Fig. 4a). A key anchoring amino acid in both proteins is Asn642, which has been shown to be mutated in some cancers^40^. To further confirm their binding in cells, we performed cellular thermal shift assays (CETSA) to assess protein stability. For this, KHOS/NP cells were incubated with pimozide for 4 h at room temperature, and subsequently subjected to increasing temperatures from 40 to 59 °C. Western blot analyses demonstrated that thermal denaturation of STAT5 in control, DMSO cells occurs at 46 °C. However, following pimozide treatment, the denaturation temperature changed to 55 °C (Fig. 5b). On the other hand, the compound did not affect the stability of GAPDH. This suggests that pimozide specifically binds and provides stabilization of STAT5 protein in the cells. We also determined whether pimozide interaction with the STAT5 affects phosphorylation, especially because the compound was found to interact with specific amino acids around the phosphorylation site. There was a significant reduction in both phosphorylation of STAT5 protein (Fig. 4c) In addition, there was a reduction in the phosphorylation of STAT3 and ERK1/2 MAP kinase following pimozide treatment (Fig. 4c). These data suggest that pimozide inhibits STAT-3 and -5 phosphorylation and its downstream signaling.

### STAT5 protects cells from pimozide-mediated cell death

Previous studies suggested that pimozide is a STAT5 inhibitor^10,42^. To determine the role of STAT5 in pimozide mediated OS cell death, we used specific silencer RNAs for STAT5A and STAT5B to downregulate these proteins in KHOS/NP cells (Fig. 5a). However, the antibodies used to detect total protein and the phosphorylated form recognize both STAT5A and STAT5B. Nevertheless, the western blot suggests that there is higher levels of STAT5B in the cells compared to STAT5A. Cell cycle analyses demonstrated that this did not significantly change cell cycle progression of the cells (Fig. 5b). However, there was about 35% reduction in proliferation at baseline in the STAT5A and STAT5B knocked down cells (Fig. 5c). Moreover, in cells treated with pimozide there was a further reduction in the proliferation of the cells, and this did not change with the levels of STAT5A or STAT5B (Fig. 5c). We next determined the effect of suppressing STAT5 expression on pimozide mediated apoptosis. While suppressing STAT5A or STAT5 did not induce cleavage mediated activation of caspase 3, treatment with pimozide following STAT5A or STAT5 knockdown significantly induced caspase 3 cleavage (Fig. 5d). This suggests that STAT5 may protect cells from pimozide induce cell death. To confirm this, we next performed flow cytometry studies. We treated KHOS/NP and SJSA-1 cells with 10 µM pimozide and subsequently determined phosphatidylserine externalization in apoptotic cells using recombinant annexin V conjugated to green-fluorescent FITC dye. In addition, to identify dead cells, we stained the cells with propidium iodide (PI). While apoptotic cells will produce green fluorescence, dead cells would give red fluorescence. Flow cytometry analyses suggested that just knockdown of STAT5A or STAT5B demonstrated no changes in cell undergoing necrosis and apoptosis (Fig. 5e). However, following pimozide treatment there was significantly increased levels of necrosis and apoptosis in KHOS/NP cells (Fig. 5e). In addition, there was also an interesting difference observed between cells where STAT5A or STAT5B was knocked down. While cells in which STAT5A was knocked down, the effects of pimozide was similar to that seen in with controls, knockdown of STAT5B resulted in reduced levels of pimozide mediated necrosis, and a corresponding increase in late apoptosis (Fig. 5e). These data suggest that STAT5B may protect the cells from pimozide mediated apoptosis.

### Pimozide inhibits growth of KHOS/NP tumor xenografts

**Fig. 6:**
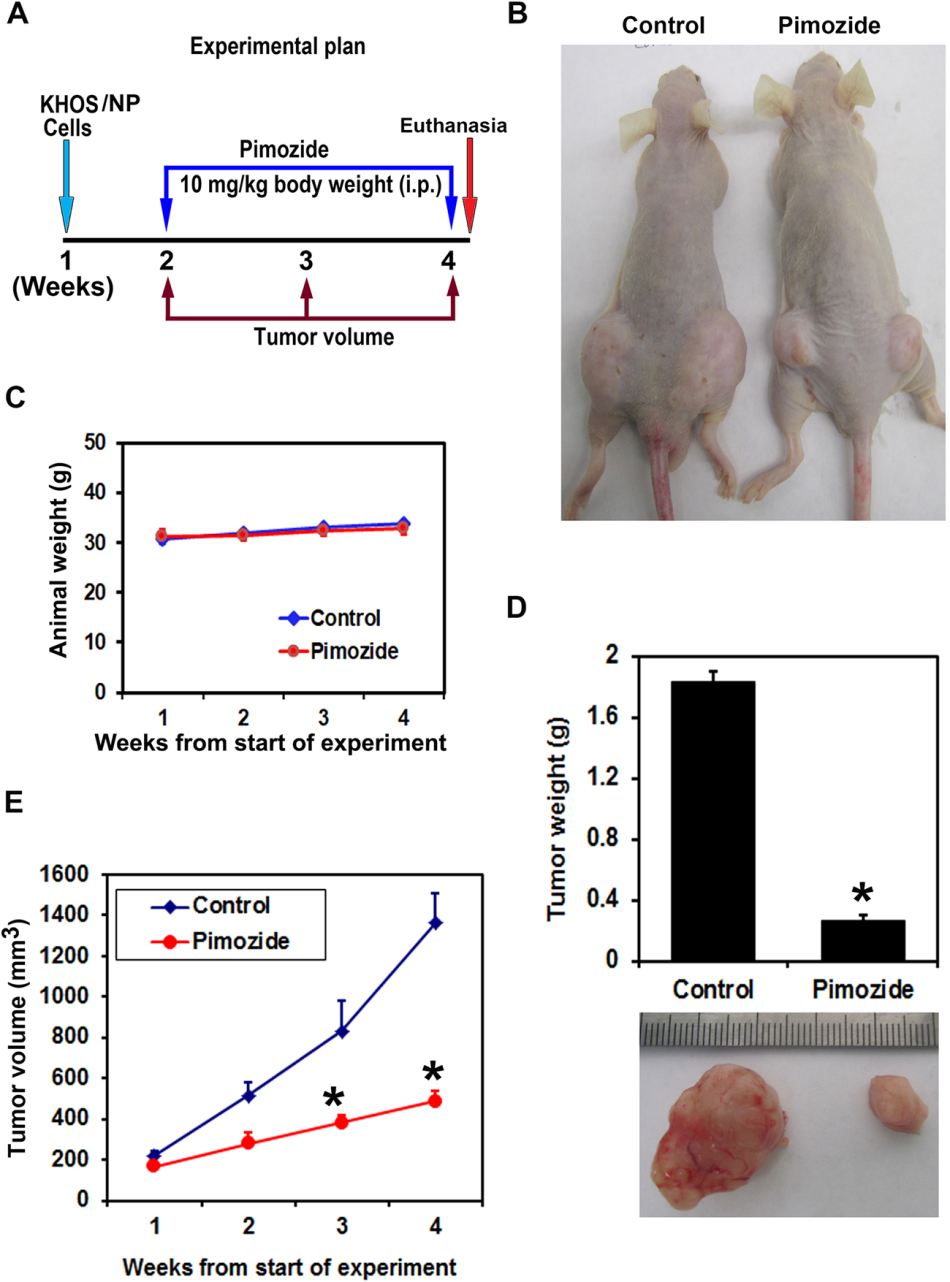
Pimozide inhibits KHOS/NP induced osteosarcoma tumor xenograft growth. **a** Experimental plan, **b** KHOS/NP cells were injected in to the flanks of nude mice and palpable tumors were allowed to develop for 7 days. Subsequently, pimozide (10 mg/kg bw) was injected daily intraperitoneally every day for 21 days. Tumor size was measured every week. On day 22, tumors were excised and subject to further analyses. **c** Pimozide treatment did not reduced the animal weight. **d** Tumor weight in pimozide treated mice were smaller when compared to control (*P < 0.004). **e** Pimozide treatment significantly reduced tumor volumes (*P < 0.001). Error bars represent means ± SEM (*N* = 5 for each group). *P < 0.05, Student’s t-test.

To evaluate the role of pimozide on tumor growth in vivo, we examined tested the compound on subcutaneous growth of OS xenografts. KHOS/NP cells were injected into the flanks of athymic nude mice, and xenograft tumors were allowed to develop. After one week, pimozide was administered intraperitoneally at a dose of 10 mg/kg bw daily for three weeks (Fig. 6a, b). There was no significant difference observed in the animal weights following treatment with pimozide (Fig. 6c). Pimozide treatment significantly inhibited the growth of tumor xenografts, resulting in reduced tumor volumes (*p* < 0.001) (Fig. 6e). The excised tumors from control animals weighed ~1700 mg, while those from animals treated with pimozide weighed ~300 mg (*p* < 0.004) (Fig. 6d). There was no apparent change in liver and spleen weight in the animals (data not shown). These data imply that pimozide is a potential therapeutic agent for treating OS with relatively non-toxic effects to the animals.

### Pimozide suppresses STAT5 signaling and OS stem cell marker expression in tumor xenograft tissues

**Fig. 7:**
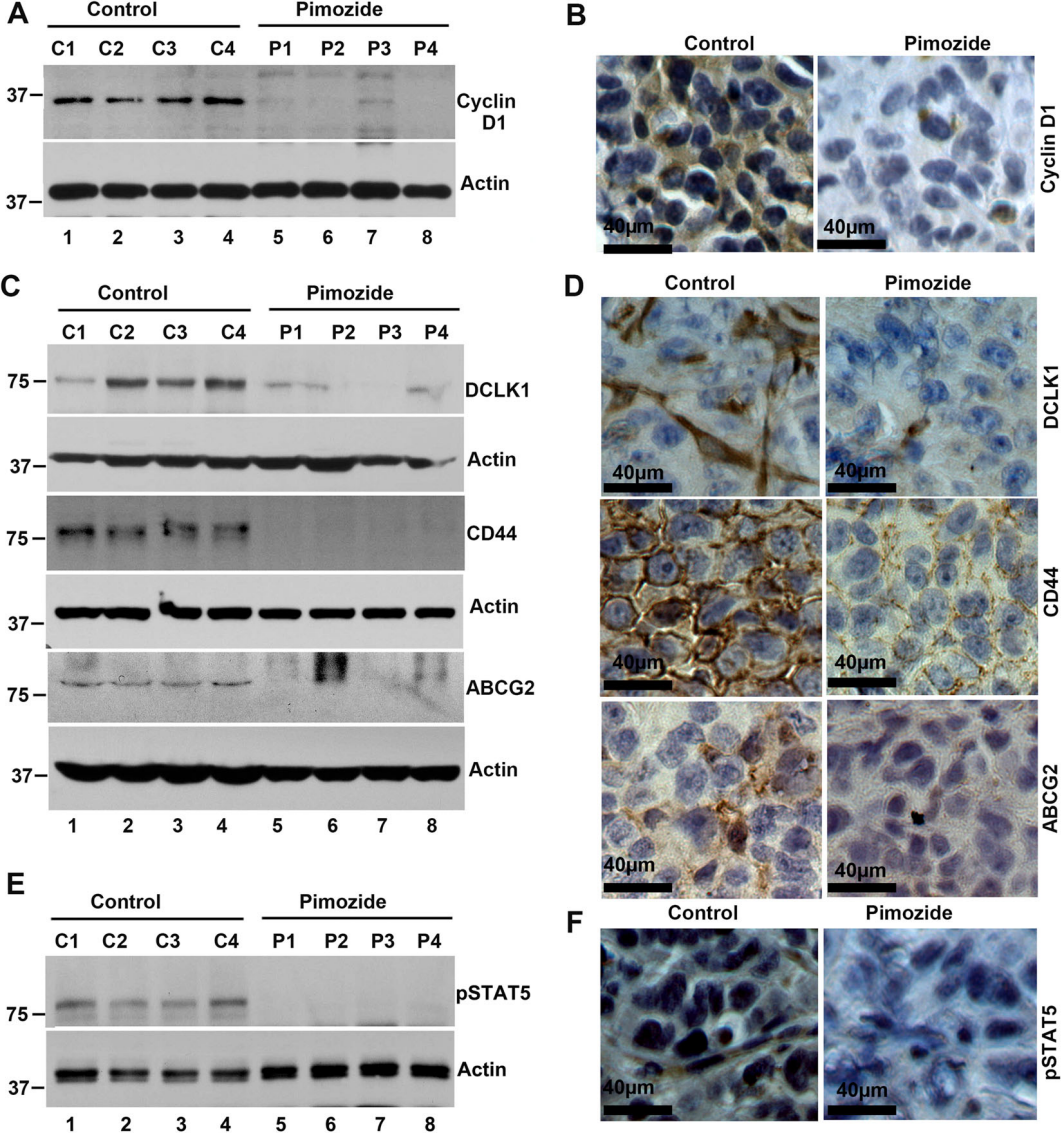
Pimozide inhibits cyclin D1, cancer stem cell markers, and STAT5 signaling proteins in tumor xenograft tissues. **a** Western blot (C1, C2, C3, C4 are control tumor xenograft tissues and P1, P2, P3, P4 are pimozide treated tumor xenograft tissues) and **b** immunohistochemistry analyses showed that tissue lysates from pimozide treated animals have significantly lower levels of cyclin D1 expression. Scale bars represent 40 μm. **c** Western blot (C1, C2, C3, C4 are control tumor xenograft tissues and P1, P2, P3, P4 are pimozide treated tumor xenograft tissues) and **d** immunohistochemistry analyses showed that tissue lysates from pimozide treated animals have significantly lower expression levels of cancer stem cell markers DCLK1, CD44 and ABCG2. Scale bars represent 40 μm. **e** Western blot (C1, C2, C3, C4 are control tumor xenograft tissues and P1, P2, P3, P4 are pimozide treated tumor xenograft tissues) and **f** immunohistochemistry analyses showed that tissue lysates from Pimozide treated tumor tissues as significantly reduced the phosphorylation of STAT5. Scale bars represent 40 μm.

Since we observed a reduction in cyclin D1 in the in vitro cell culture studies, we determined the effect of pimozide treatment on cyclin D1 expression in the xenograft tissues. Western blot and immunohistochemistry analyses demonstrated that pimozide treatment significantly reduced cyclin D1 levels when compared to controls (Fig. 7a, b). Next, we determined whether pimozide affected OS stemness by looking for expression of specific stem cell marker proteins. Both western blot and immunohistochemistry analyses demonstrated that pimozide treatment significantly reduced the levels of OS stem cells marker proteins DCLK1, CD44, and ABCG2 when compared to controls (Fig. 7c, d). These data suggest that pimozide targets OS stem cells with high potency. Furthermore, we observed a significant reduction in STAT5 phosphorylation in the xenograft tissues (Fig. 7e, f). These data suggest that pimozide inhibits cell proliferation, as well as OS stem cells in the tumor xenograft tissues.

## Discussion

STAT5 has been shown to be a key player in tumor cell survival, proliferation and invasion^43^. However, although suppressing JAK/STAT signaling appears to be a promising strategy in inhibiting tumor growth, targeting the protein has been rather difficult. Small molecules have been developed that targets the SH2 domain including pimozide, which has shown some promise against acute myelogenous leukemia^42^. Given the high STAT5 activity in OS cells, we tested the effect of the compound and see high activity. Pimozide is an antipsychotic FDA-approved drug used to treat neuroleptic disorders. Pimozide had relatively low side-effect and exhibits a broad spectrum of molecular targets including monosymptomatic hypochondriacal psychoses, body dysmorphic disorder, metastatic melanoma, trichotillomania, and trigeminal and postherpetic neuralgia^44^. Pimozide has been shown to inhibit the growth of various cancer cells including prostate cancer, lung, breast, colon, liver, glioblastoma and leukemia^6,45^. These studies suggest that pimozide could be used as an effective chemotherapeutic agent. Our results also indicate that the pimozide has a significant potential in the treatment of OS.

Previous studies have suggested that pimozide is a STAT5 inhibitor although the mechanism by which the inhibition works is not well understood^42^. Our studies demonstrate that pimozide interacts with Asn642. Mutations at Asn642 is a common oncogenic driver mutation that promotes aggressive T-cell leukemia/lymphoma^40^. Since pimozide binds to this site in STAT5B, suggests that masking this site may be important to suppress the protein function. Similarly, it is interesting that STAT5B may protect the cells by sequestering the compound, and knockdown of the protein therefore enhances pimozide activity against the cell. This might one reason for why targeting the protein in cancer cells have been difficult.

Pimozide appears to inhibit many of the characteristic cancer-promoting events. Previous studies demonstrated that pimozide inhibits cell proliferation and induces ROS generation in human OS by suppressing catalase expression^36^. Previously, we have suggested that natural compounds targeting major cell signaling pathways may be a novel paradigm for OS therapy including Wnt, Notch, Hedgehog, Hippo, and JAK-STAT signaling pathways^25,46^. CSCs have the capacity to drive tumor resistance and recurrence to chemotherapeutic agents^37,47^. Pimozide has been previously shown to suppress stem-like cell maintenance and tumorigenicity in hepatocellular carcinoma^36^. Indeed, our results suggest that the pimozide is a potent inhibitor of OS stem cells. The compound suppressed expression of known OS stem cell markers CD44, CD133, Oct-4, and ABCG2, both in cells grown as 2D cultures and in osteospheres. Moreover, we have now identified that DCLK1, a reserve quiescent stem cell marker in both the normal intestinal epithelium and in colon cancers^18,19^ is also expressed in OS cells. More importantly, the short variant of DCLK1 is upregulated in osteospheres, and this is suppressed in response to pimozide. Hence, targeting these cells with pimozide appears to be a promising approach for the treatment of OS.

In conclusion, the current studies provide evidence that pimozide is able to suppress the growth of OS stem and progenitor cells, and that this may in part through suppressing STAT5 activity. Hence, the compound or its derivatives may be of interest as a therapeutic for OS either alone or in combination with current standard therapeutics. Additional studies are required as a follow up to the studies presented in this manuscript to further move the compound or its analog to the clinic for the treatment of patients with OS.

## Methods

### Cell lines and reagents

KHOS/NP, MG63, and SJSA-1 human OS cells (American Type Culture Collection, Manassas, VA) were grown in DMEM containing 10% heat-inactivated fetal bovine serum (Sigma-Aldrich, St. Louis, MO, USA) and 2% antibiotic-antimycotic solution (Corning Life Sciences, Oneonta, NY, USA) at 37 °C in a humidified atmosphere containing 5% CO_2_. The studies were performed with cells within 20 passages after receipt or resuscitation (~3 months of non-continuous culturing). We authenticated the cell lines by STR allele profiling by an independent source (NIH-funded University of Arizona Genetics Core, Tucson, AZ-Cell line Authentication Core). Eighty percent homology demonstrates validity of the cell lines. We tested cell lines for mycoplasma. Pimozide was obtained from Sigma-Aldrich and dissolved in dimethyl sulfoxide (DMSO).

### Proliferation assay

To assess proliferation, the cells were seeded on to 96 well plates and grown overnight. Thereafter, the cells were treated with increasing doses of pimozide (0–40 µM) in DMEM containing 10% FBS. Analysis of cell proliferation was performed by the enzymatic assay as previously described^48^.

### Colony formation assay

Briefly, 6 well dishes were seeded with 500 viable cells and allowed to grow for overnight. Thereafter, the cells were incubated in the presence or absence of 0–10 µM Pimozide for 48 h. Pimozide containing medium was then removed, and the cells were washed in PBS and incubated for an additional 10 days in complete DMEM medium. Each treatment was done in triplicate. The colonies obtained were washed with PBS and fixed in 10% formalin for 15 min at room temperature and then washed with PBS followed by staining with Crystal violet. The colonies were counted and compared with the untreated cells.

### Tumor in A Dish (TiD)

1000–2000 cells each of normal human lung epithelial cells, normal human lung fibroblasts, human umbilical vein endothelial cells and normal lymphatic endothelial cells were mixed with KHOS/NP GFP positive cells and grown in an ultra-low attachment using specific spheroid media^27^. Once the organoid had developed, it was treated with Pimozide. After 5 days of treatment, the organoids were assessed by Nikon Eclipse Ti microscope under a ×40 objective. Subsequently, the cells were separated and the number of GFP + KHOS/NP cells were determined by flow cytometry. Flow cytometry was done with a FACSCalibur analyzer (Becton Dickinson, Mountain, View, CA, USA), capturing 10,000 events for each sample. Results were analyzed with ModFit LT™ software (Verity Software House, Topsham, ME).

### Cell cycle analyses

Cells were treated with 20 µM of pimozide for up to 48 h. Thereafter, the cells were trypsinized and suspended in phosphate-buffered saline (PBS). Single-cell suspensions were fixed using 70% ethanol for overnight, and subsequently permeabilized with PBS containing 1 mg/mL propidium iodide (Sigma-Aldrich), 0.1% Triton X-100 (Sigma-Aldrich) and 2 µg DNase-free RNase (Sigma-Aldrich) at room temperature. Flow cytometry was done as mentioned above.

### Caspase3/7 assay

KHOS/NP and SJSA-1 cells grown in 96 well black plates were treated with pimozide for up to 48 h. Caspase3/7 activity was examined using the Apo-ONE® Homogeneous Caspase-3/7 Assay systems according to the manufacturer’s instructions (Promega, Madison, WI).

### Western blot analysis

Cell lysates were subjected to polyacrylamide gel electrophoresis, and then blotted onto Immobilin-P polyvinylidene difluoride membranes (Millipore, Bedford, MA). Antibodies for cyclin D1 (catalog # 2978), CDK2 (catalog # 2546), phospho-Rb (catalog # 8516), Rb (catalog # 9309), PARP(catalog No # 9532), Caspase 3 (catalog # 9662), Bcl2 (catalog # 2876), BclXL (catalog # 2764), Bax (catalog No # 5023), CD44 (catalog # 3570), CD133 (catalog # 64326), phospho-STAT5 (catalog #s 4332, 9359), STAT5 (catalog # 25656), phospho-STAT3 (catalog # 9131), STAT3 (catalog # 4904), phospho-ERK (catalog # 4370) and ERK (catalog # 9102), ABCG2 (catalog #42078), DCLK1 (catalog # 62257) were purchased from Cell Signaling Technology (Danvers, MA, USA), for DCLK1 (catalog # ab37994), Oct-4 (catalog # ab189857) from Abcam Inc. (Cambridge, MA, USA), CDK4 (catalog # MA5–13498), CDK6 (catalog # MA5–13338) from Thermofisher (Waltham, MA, USA) and DCLK1 (catalog # SAB2420186) and Actin (catalog # A1978) from Sigma Aldrich and GAPDH (catalog # sc-365062) Santacruz Biotechnology Inc (Santa Cruz, CA, USA). Specific proteins were detected by chemiluminescence system.

### Spheroid assay

Cells were cultured in DMEM supplemented with 20 ng/mL bFGF (Invitrogen) 10 mL per 500 mL of 50× B27 supplement (Invitrogen) EGF 20 ng/mL (Invitrogen) and antibiotics and antimycotic solution. Cells were seeded at low densities (2000 cells/mL) in 24 well low adhesion plates. Thereafter, the cells were treated with increasing concentrations of pimozide (0–25 μM). After 5 days the spheroids were photographed. For second passages, the primary spheroids were dissociated with trypsin, and cells were grown in the absence of pimozide.

### Molecular docking

The docking study was performed by using AutoDock Vina software^49^ to study the interaction of Pimozide with the crystal structure of STAT5 extracted from Protein Data Bank (STAT5A (PDB:1Y1U) and STAT5B (PDB:6MBW)) online database (www.rcsb.org/pdb)^50^. The ligand molecules in the active site were removed and the 3-D grid box was created with grid center coordinates and 60 × 60 × 60 point size covering all active site residues within it. All the docking studies were executed based on default parameters of the Autodock Tools. Prior to docking, the protein was prepared by adding hydrogens, total Kollman and Gasteiger charges. Lamarckian GA was used to search the best conformations. About 10 conformations for pimozide docked in the SH2 domain of STAT5A or STAT5B were generated. Further, the most stable conformation of pimozide was selected based on a predicted score by the scoring function and the lowest binding energy. The STAT5 docking conformation was visualized with Pymol^51^.

### Cellular thermal shift assay (CETSA)

The ability of pimozide to interact with STAT5 in cells was studied by CETSA^52^. Briefly, KHOS/NP cells were cultured and grown to 70–80% confluency. Cells were detached with trypsin, collected by centrifugation, washed with PBS and subsequently resuspended in DMEM media for counting. The cell suspension is treated with media containing DMSO or pimozide (20 μM) for 4 h. After treatment, the cell suspension was aliquoted into PCR tubes and heated for 3 min at different temperature gradient. Subsequently, cells were lysed using two repeated freeze-thaw cycles using liquid nitrogen followed by centrifugation for 20 min. The resultant proteins were diluted with 4× Laemmli buffer, boiled at 70 °C for 10 min and loaded onto 10% SDS-PAGE gel, transferred to PVDF membrane and incubated with STAT5 antibody from Cell Signaling at a concentration of 1:500. Protein levels on western blot were pictured by Bio-Rad ChemiDoc-XRS + instrument and analyzed by image lab software.

### siRNA

STAT5A siRNA ID s13534 (locus ID 6776) sequence was ACAGAACCCUGACCAUGUAtt, STAT5B siRNA ID s13538 (locus ID 6777) sequence was CACCCGCAAUGAUUACAGUtt and a scrambled control siRNA not matching any of the human genes were obtained from ThermoFisher Scientific, USA and transfected using siPORT™ NeoFX™ Transfection Agent (ThermoFisher Scientific, USA). Briefly, KHOS/NP cells were split and added to the 96 or 6 well plates and immediately transfected with 100 nM siRNA and after 72 h measured for cell proliferation or 48 h measured for cell cycle analysis. After 36 h the KHOS/NP or SJSA-1 cells were treated with pimozide for 24 h. Subsequently, western blot analysis or Annexin V/FITC flow cytometry was performed.

### Annexin V/PI staining

Briefly, KHOS/NP cells were split and added to the 6 well plates and immediately transfected with 100 nM siRNA. After 36 h the cells were treated with pimozide for 24 h. Thereafter, the cells were trypsinized and suspended in ice cold phosphate-buffered saline (PBS). For Annexin V/PI staining, flow cytometry was performed using dead cell apoptosis kit with Annexin V FITC and PI solution (Thermo Fisher Scientific, Waltham, MA) as per the manufacturer instructions. Results were analyzed with FlowJo software.

### KHOS/NP xenograft tumors in mice

Seven-week-old male athymic nude mice (*N* = 5 per group), purchased from Charles Rivers Laboratory were utilized for in vivo experiments. They were maintained with water and standard mouse chow ad libidum and used in protocols approved by the University’s Animal Studies Committee. Animals were injected with 1 × 10^6^ KHOS/NP cells in the left and right flank and allowed to form xenograft. One week following implantation, and after observing the presence of a palpable tumor, pimozide (10 mg/kg body weight mixed with 25 mM phosphate buffer in 50 mM captisol) was administered intraperitoneally daily for 21 days. Tumors were measured weekly by blinded observer. At the end of treatment, the animals were euthanized, and the tumors were removed, weighed and used for histology, immunohistochemistry, and western blot analysis.

### Immunohistochemistry

Paraffin-embedded tissues were cut to 4 µm sections, deparaffinized and blocked with Avidin/Biotin for 20 min. The slides were incubated with primary antibodies for overnight at 4 °C. Next the slides were treated with broad-spectrum secondary antibody (Invitrogen) and HRP-conjugate for 1 h and then developed with DAB (Invitrogen). Finally, the slides were counterstained with hematoxylin. The slides were examined in Nikon Eclipse Ti microscope under a ×40 objective.

### Statistical analysis

All values are expressed as the mean ± SEM. Data was analyzed using an unpaired 2-tailed *t* test. A *P* value of less than 0.05 was considered statistically significant.

## Supplementary information

Supplementary Figure Legends

Supplementary Figure 1
