# Supplementary material for: Suppressing STAT5 signaling affects osteosarcoma growth and stemness
Source: Cell Death Dis. 2020 Feb 24;11(2):149. doi: 10.1038/s41419-020-2335-1 (PMC7039889; doi:10.1038/s41419-020-2335-1)
Supplement: Supplementary file 1 — Supplementary Figure Legends [file 41419_2020_2335_MOESM1_ESM.docx]

**Supplementary Fig. 1: Pimozide induces cell cycle arrest and apoptosis**

**a** Cells were incubated with pimozide 10 and 20 µM concentration for 48 h and assessed for apoptosis by Caspase3/7 assay. Pimozide treatment results in significant increase in Caspase3/7 activity in both KHOS/NP and SJSA-1 cells (*p<0.001).

**b** Cells were treated with 20μM of pimozide for 24 and 48 h, the lysate was analyzed by western blotting for CDK4 and CDK6 expression. Pimozide treatment inhibits CDK4 and CDK6 expressions in both KHOS/NP and SJSA-1 cells.
