# Supplementary figures and images for: Suppressing STAT5 signaling affects osteosarcoma growth and stemness
Source: Cell Death Dis. 2020 Feb 24;11(2):149. doi: 10.1038/s41419-020-2335-1 (PMC7039889; doi:10.1038/s41419-020-2335-1)

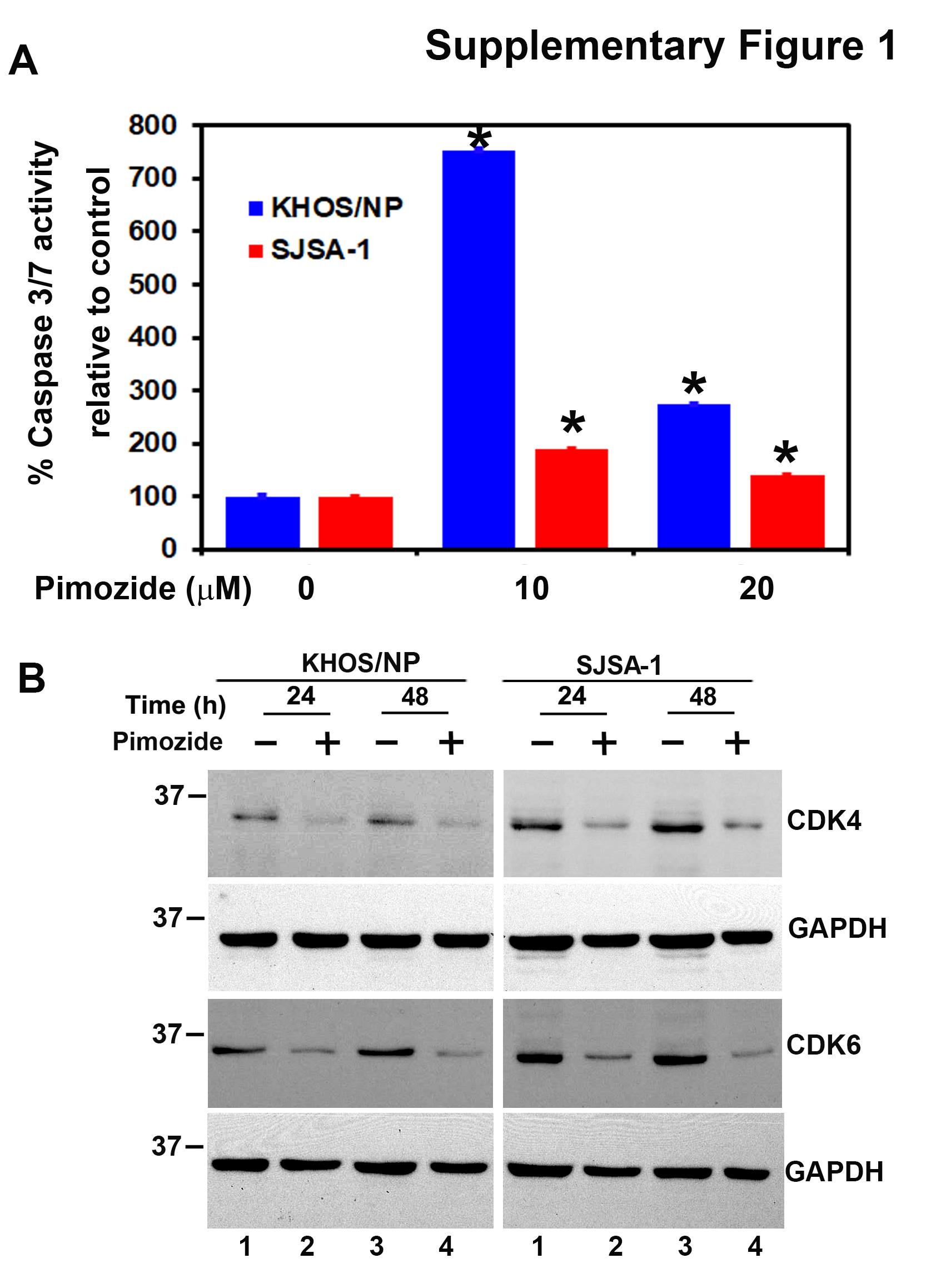

Supplement: Supplementary file 2 — Supplementary Figure 1 [file 41419_2020_2335_MOESM2_ESM.tif]
